# Preoperative anxiety predicted the incidence of postoperative delirium in patients undergoing total hip arthroplasty: a prospective cohort study

**DOI:** 10.1186/s12871-021-01271-3

**Published:** 2021-02-12

**Authors:** Jun Ma, Chuanyao Li, Wei Zhang, Ling Zhou, Shuhua Shu, Sheng Wang, Di Wang, Xiaoqing Chai

**Affiliations:** 1 Anhui Provincial Hospital, Cheeloo College of Medicine, Shandong University, Jinan, 250012 Shandong China; 2 Department of Anesthesiology, The First Affiliated Hospital of USTC, Hefei, 230001 Anhui China

**Keywords:** Anxiety, Delirium, Cognitive ability, Hip surgery

## Abstract

**Background:**

Delirium was characterized with a series of symptoms of a sudden onset of disturbances in attention, a loss in memory loss and defects in other cognitive abilities that were also appeared in the syndrome of anxiety. Even though there are overlapped clinical symptoms existed in anxiety and delirium, the relationship between anxiety and delirium was still unclear. The propose of this study was to investigated the effect of preoperative anxiety on postoperative delirium.

**Methods:**

Three hundred and seventy-two adults undergoing total hip arthroplasty were enrolled from October 2019 to May 2020 in the study. The preoperative anxiety was measured with the Hospital Anxiety and Depression Scale-Anxiety (HADS-A). The participants were allocated into anxiety group (HADS-A≧7) and non-anxiety group (HADS-A < 7). The primary outcome was the incidence of the postoperative delirium assessed with the Confusion Assessment Method (CAM). The secondary outcomes were the duration and the severity of delirium evaluated with the Memorial Delirium assessment Scale (MDAS). The risks of delirium were also evaluated with logistic regression analysis.

**Results:**

There were 325 patients enrolled in the end, 95 of whom met the criteria for anxiety. The incidence of delirium was 17.8% in all participants. The patients with anxiety had a higher incidence of delirium than the non-anxiety patients (25.3% vs. 14.8%, odds ratio (OR) = 0.51, 95% confidence interval (CI) = 0.92–0.29, *p* = 0.025). However, no significant differences were found in the duration and the severity of the delirium between the above two groups. The age, alcohol abuse, history of stroke, scores of the HADS-A, and education level were considered to be predictors of delirium.

**Conclusions:**

The preoperative anxiety predicted the incidence of the postoperative delirium in total hip arthroplasty patients. The related intervention may be a good point for delirium prophylaxis.

**Trial registration:**

It was registered at Chinese Clinical Trial Registry (www.chictr.org.cn) with the name of “the effect of preoperative anxiety on the postoperative cognitive function” (ChiCTR1900026054) at September 19, 2019.

## Background

The postoperative delirium (POD), defined as a sudden onset of disturbances in attention, consciousness and other cognitive abilities, was one of the common surgical complications with bad outcomes. It not only had a closed relationship with other postoperative complications, such as the cognitive impairment, the high incidence of the death after surgery [1, 2], but also increased the burden of the community, including high expensive health expense and more medical resources [3–6]. Although the underling pathogenesis mechanism of delirium is ambiguous, it is of great significance to study the risk factors associated with the POD to moderate its consequences.

Many risk factors for the delirium [7–20] were reported by previous studies, including age, the educational level, anemia, operative time, alcohol abuse, physical function, medications, blood loss, infection, and cognitive function.

All the risks described above had the same character --it is hard for them to be altered by interventions that may be potentially preventive for the POD. It is more meaningful to find some risk factors that are not only specific for high-risk groups but also can be interfered for prevention. For this reason, psychiatric symptoms were a good indicator. As they had a close relation with the metabolism and function of nervous system [17, 18]. It was proved that depression was a risk factor for the POD [11, 12, 15–19, 21]. Anxiety was another psychiatric symptom in the clinical practice, which was quite common before surgery. There were several studies related to anxiety [17–19, 22], however, the relationship between the preoperative anxiety (POA) and the POD remained unclear. Thus, this issue needed further clarification.

Orthopedic surgery, especially hip surgery in elderly, has the high incidence of POD [23, 24], most likely due to advanced age, preoperative cognitive impairment and multiple comorbidities among these patients [25]. A total of 5–45% of orthopedic surgery patients experienced delirium [26–28]. Therefore, we planned a prospective study to clarify the relationship between the POA and the POD via orthopedic surgery. The aim of the study was to investigate whether the POA would predict the onset of POD in patients undergoing the total hip arthroplasty (THA).

## Methods

### Setting and subjects

Ethical approval for this study [2019-N(H)-100] was provided by the biomedicine ethics board of the University of Science and Technology of China (USTC), Hefei, China (Chairperson Professor Liu) on 5 March 2019. The study was registered at the Chinese Clinical Trial Registry (ChiCTR) with the number of ChiCTR1900026054. We consecutively recruited people aged 18 years or older, ASA I-III, undergoing the THA at the Anhui Provincial Hospital (AHPH) from October 2019 to May 2020. We excluded individuals who (1) were unable to provide written informed consent, (2) were not fluent in Chinese, (3) had a history of depression or diagnosed with depression (assessed with the Hospital Anxiety and Depression Scale-Depression [15] and diagnosed by psychiatrists), (4) had dementia or scored 24 or lower on the Mini-mental State Examination (MMSE) [15, 29], or (5) had a score of 15 or higher on the Alcohol Use Disorders Identification Test [30, 31], (6) not evaluated the level of anxiety, (7) not completed the follow-up assessment of delirium. The informed consent was written by all participants.

### Demographic and clinical characteristics

We collected information about participant demographics (age, sex, BMI, and education), lifestyle (alcohol abuse, cigarette smoking). And the clinical characteristics were obtained from the clinical medical chart, including surgery sites and co-morbidity.

### Preoperative anxiety

The anxiety was assessed using the Hospital Anxiety and Depression Scale-Anxiety (HADS-A), as it was described before [32–34]. Briefly, it was a self-reported instrument consisting of 7 items and a 4 points Likert-scale with score ranged from 0 to 21. The severity of anxiety increased in participants with high scores in the HADS-A test. The cut-off point of HADS-A was 6/7 according to the previous study [34]. The participants with scores less than 7 were grouped in the non-anxiety group. And those with score at 7 and more were diagnosed as clinical anxiety and allocated into the anxiety group. The participants were blinded to the criteria and the grouping information.

### Postoperative delirium

The incidence of delirium was the primary outcomes of the trial. Participants were assessed twice daily (in the morning and afternoon) during the first 7 postoperative days by trained researchers and nurses who administered the Confusion Assessment Method (CAM). The assessment included a review of the patients file and discussion with their allocated ward nurse and a close relative (if available). The participants who screened positive for delirium were assessed by an experienced geriatric psychiatrist to confirm. The researchers and nurses conducted the assessment of delirium after surgery were blinded to the information about grouping. The CAM is a widely used and well-validated screening tool for delirium [35–37], with sensitivity of 94% (95% confidence interval [CI] = 91–97%) and specificity of 89% (95% CI = 85–94%) and has been successfully adapted for use in the intensive care unit (ICU) setting (the CAM-ICU was used where appropriate) [38].

### Other risk factors of POD

Secondary measures of interest included the difference between groups in the duration and severity of delirium as well as length of stay (LOS) and cognitive function. The Memorial Delirium Assessment Scale (MDAS) was performed to assess the severity of delirium [39] and was administered daily if delirium was present (range = 0–30). The duration of delirium and LOS were measured by the number of days.

### Procedure

We consecutively recruited participants who were admitted to the AHPH for the THA. The demographic data, life style, clinical characteristics, MMSE were recorded on admission by the trained research assistant with patients interview and chart review. Then we assessed the level of the anxiety in the afternoon of the day before surgery with the HADS-A [22]. The general anesthesia was performed in all participants. The surgical procedures were provided by one surgical team to avoid the bias from surgical procedures. The surgeons and anesthesiologists were blinded to the information about grouping. The assessment of the delirium was performed on the first 7 days after surgery as well as the duration and the severity. In participants with the development of the delirium, the severity was assessed using the MDAS and the duration was measured by days.

### Statistical analysis

On the basis of the previous study [40] report that postoperative delirium occurred in 1.18% in age 50s of patients after orthopedic surgery, and given the prevalence of delirium of 13% which was the highest rate in the study. With significance and power set at 0.05 and 90% respectively, the sample size of 94 patients was required to detect differences in each group. Of 95 patients in the anxiety group and 230 patients in the non-anxiety group were enrolled in the study.

The software of IBM SPSS statistics 25 was used to manage and analyze the data. Descriptive statistics were used to summarize data according to the allocation. Missing data from the cases not assessing anxiety and delirium were deleted from the analysis. We performed Student’s *t* test for normally distributed data, the Mann-Whitney U test for ordinal data without a normal distribution, and χ2 tests for proportion distributions to compare the anxiety group and non-anxiety group. *p* value < 0.05 was considered statistically significant.

Variables that achieved significance on uni-variate logistic regression analysis were entered into multivariate logistic regression analysis to estimate the risk of postoperative delirium. The multiple logistic regression analysis was used to adjust for multiple risk factors and interactions. Odds ratio (OR) and 95% confidence interval (CI) were presented to evaluate risk factors.

## Results

### Samples

**Fig. 1:**
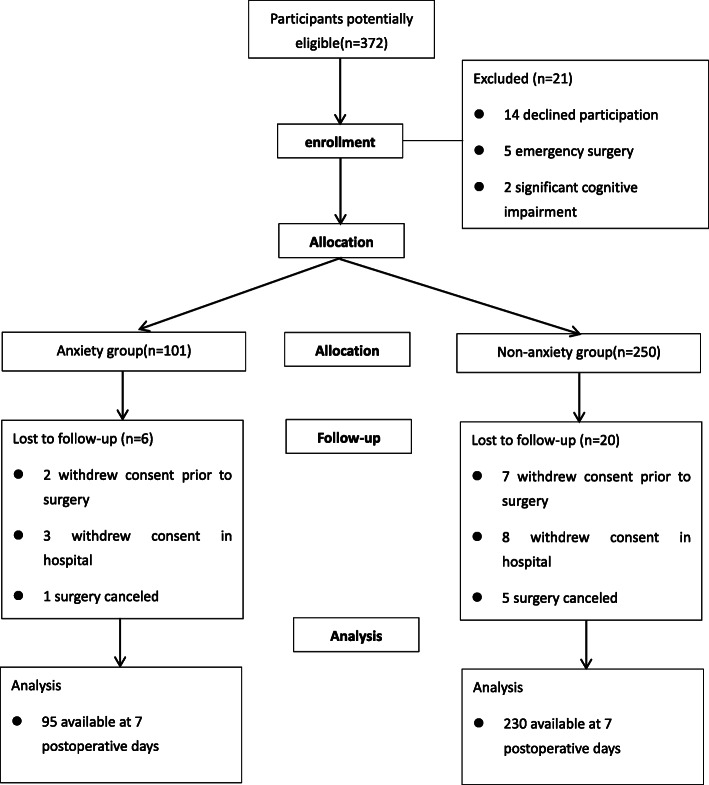
Flow of participants in the trial

A total of 372 individuals undergoing the THA enrolled in the study. They were allocated into the anxiety group and non-anxiety group according to the HADS-A test except 21 people (14 declined participation, 5 emergency surgery, 2 significant cognitive impairment). Six people in the anxiety group and 20 in the non-anxiety group withdrew consent or lost to follow-up during the study. Finally, there were 325 participants available at 7 postoperative days, including 95 in the anxiety group and 230 in the non-anxiety group (Fig. 1). The follow-up time was ended at 7 June 2020 due to the cognitive assessment at 7th postoperative day.

### Baseline characteristics

The prevalence of the anxiety in patients undergoing the THA was 29.2% of all 325 participants. The baseline characteristics of the participants, including demographic data, life style, and clinical characteristics were shown in Table 1. No differences were found in all variables in the Table 1.

**Table 1:** Baseline characteristics of 325 adults allocated to anxiety or non-anxiety groups

Characteristic	Anxiety group (***n*** = 95)	Non-anxiety group (***n*** = 230)	***p*** value
**Demographic data**			
Age in yrs., mean (SD)	52.1(13.8)	52.3(13.6)	0.905
Male, n(%)	60(63.2%)	124(53.9%)	0.126
BMI in kg/m^2^, mean (SD)	27.9(3.6)	27.2(3.1)	0.079
Education, completed high school or better, n(%)	38(40.0%)	77(33.5%)	0.263
**Lifestyle**			
Alcohol abuse, n(%)	10(10.5%)	20(8.7%)	0.604
Ever smoked, n(%)	27(28.4%)	70(30.4%)	0.718
Current smoked, n(%)	33(34.7%)	68(29.6%)	0.360
**Clinical characteristics**			
History of stroke, n(%)	16(16.8%)	43(18.7%)	0.693
IHD, n(%)	37(38.9%)	84(36.5%)	0.681
Hypertension, n(%)	42(44.2%)	95(41.3%)	0.629
Diabetes, n(%)	34(35.8%)	74(32.2%)	0.529
Which side of total hip arthroplasty, n(%)			0.796
left	46(48.4%)	115(50.0%)	
right	49(51.6%)	115(50.0%)	
ASA, n(%)			0.803
I	8(8.4%)	15(6.5%)	
II	19(20.0%)	44(19.3%)	
III	68(71.6%)	171(74.3%)	
MMSE before surgery, mean (SD)	27.5(1.9)	27.1(2.2)	0.122
NSAIDs, n(%)	75(78.9%)	167(72.6%)	0.233

The two groups were allocated according to the HADS-A (people of the HADS-A ≥ 7 in the anxiety group and people of the HADS< 7 in the non-anxiety group)

*Abbreviations*: *BMI* Body Mass Index, *IHD* Ischemic Heart Disease, *ASA* American Society of Anesthesiologists, *MMSE* Mini-Mental State Examination, *SD* Standard Deviation, *NSAIDs* Non-steroidal Anti-inflammatory Drugs

### Primary outcomes

The incidence of the POD was 19.6% of all 325 participants. It was higher in patients with anxiety than those without clinical anxiety (25.3% vs. 14.8%, OR 0.51, 95% CI 0.92–0.29, *p* = 0.025), indicating that the factor of preoperative anxiety predicted the incidence of delirium (Table 2).

**Table 2:** Primary and secondary outcomes: complete case analysis

Primary Outcome	All enrolled patients (***n*** = 325)	Anxiety group (***n*** = 95)	Non-anxiety group (***n*** = 230)	***p*** value
Incidence of delirium, n(%)	58/325(17.8%)	24/95(25.3%)	34/230(14.8%)	0.025
**Secondary Outcome**	**Delirium cases (*n*** **= 58)**	**Anxiety** **(*n*** **= 24)**	**Non-anxiety (*n*** **= 34)**	
Duration of delirium> 2 days, n(%)	19(32.8%)	9(37.5%)	10(29.4%)	0.518
Severity of delirium≧13, n(%)	28(48.3%)	10(41.7%)	18(52.9%)	0.397
Patterns of delirium				0.334
hyperactive, n(%)	16	5	11	
hypoactive, n(%)	32	16	16	
Mix, n(%)	10	3	7	
Age in yrs., mean (SD)	60.5(10.6)	60.8(11.5)	60.2(10.1)	0.834
Male, n(%)	33(56.9%)	14(58.3%)	19(55.9%)	0.853
BMI i n kg/m^2,^ mean (SD)	28.0(3.7)	28.5(3.7)	27.6(3.8)	0.373
Education, completed high school or better, n(%)	9(15.5%)	5(20.8%)	4(7.0%)	0.568
MMSE before surgery, mean (SD)	27.5(2.0)	27.9(1.7)	27.1(2.1)	0.129
VAS, mean (SD)	2.4(1.5)	2.1(1.2)	2.6(1.6)	0.201
Surgery time in min, mean (SD)	81.2(31.4)	88.3(31.8)	76.3(30.6)	0.153
LOS in days, mean (SD)	7.0(2.4)	7.8(3.0)	6.4(1.6)	0.025

*Abbriviations*: *BMI* Body Mass Index, *MMSE* Mini-Mental State Examination, *VAS* Visual Analogue Scale, *LOS* Length of Stay, *SD* Standard Deviation

### Secondary outcomes

The duration and severity of the POD had no statistical differences between two groups (*p* = 0.518 and *p* = 0.397, respectively). However, the LOS were longer in the POD patients with anxiety than the POD patients without anxiety [7.8(3.0) vs. 6.4(1.6), *p* = 0.025]. No differences were found in the other variables between two groups (Table 2).

### Postoperative outcomes in all participants

Table 3 showed the postoperative variables in all participants of the study. The LOS was significantly longer in the anxiety participants than those without clinical anxiety (*p* = 0.038), suggesting that the POA might prolong the recovery time from the surgery due to the longer length of stay. There were no statistical differences in the other variables between those two groups, including admitting to ICU, transfusion, surgery time, and other postoperative complications.

**Table 3:** Postoperative outcomes in two groups

Variables	Anxiety group (***n*** = 95)	Non-anxiety group (***n*** = 230)	***p*** value
**Transfusion, n(%)**	12/95(12.6%)	32/230(13.9%)	0.759
**ICU, n(%)**	8/95(8.4%)	33/230(14.3%)	0.588
**Hypotension, n(%)**	10/95(10.5%)	12/230(5.2%)	0.083
**Hematoma, n(%)**	2/95(2.1%)	7/230(3.0%)	0.639
**Nausea, n(%)**	20/95(21.1%)	60/230(26.1%)	0.338
**Vomiting, n(%)**	9/95(9.5%)	38/230(16.5%)	0.100
**Hypothermia, n(%)**	11/95(11.6%)	16/230(7.0%)	0.170
**VAS,mean (SD)**	2.7(1.6)	2.9(1.7)	0.445
**Surgery time, min,** **mean (SD)**	75.4(29.0)	80.0(33.7)	0.239
**LOS,mean (SD)**	6.8(2.6)	6.2(1.8)	0.038

Hypotension, the blood pressures were low above 30% of the baseline or need constrictor support;

Hematoma, the data collected from postoperative clinical records or re-operation for hemostasis;

*VAS* Visual Analogue Scale, which was assessed at the first postoperative day, *MMSE* Mini-Mental State Examination, *LOS* Length of Stay, *SD* Standard Deviation

### Delirium risk factors

The logistic regression model was performed to find the predictors of the POD. The age, alcohol abuse, history of stroke, scores of the HADS-A, and education level were considered to be the predictors of the POD, identifying by a multivariate logistic regression model. Surgery time, BMI, and VAS scores were not statistically significant in this model (Table 4).

**Table 4:** Results of the multivariable logistic regression model predicting postoperative delirium

Variables	All participants (***n*** = 325)	OR(95%CI)	***p*** valve
**Surgery time, mean (SD)**	78.7(32.4)	1.003 (0.993–1.012)	0.573
**Age, mean (SD)**	52.2(13.7)	1.041(1.009–1.074)	0.012
**BMI, mean (SD)**	27.4(3.3)	1.022(0.922–1.133)	0.681
**Alcohol abuse, n(%)**	30(9.2%)	3.414(1.299–8.975)	0.013
**History of stroke, n(%)**	59(18.2%)	2.330(1.079–5.032)	0.031
**Scores of the HADS-A, mean (SD)**	6.6(3.2)	1.104(1.010–1.208)	0.030
**Education level, n(%)**	115(35.4%)	0.399(0.170–0.935)	0.034
**VAS, mean (SD)**	2.8(1.7)	0.976(0.808–1.178)	0.799

*Abbreviations*: *BMI* Body Mass Index, *VAS* Visual Analogue Scale, *HADS-A* the Hospital Anxiety and Depression Scale-Anxiety

## Discussion

All participants undergoing the THA were assessed with the HADS-A test in the trial. The result showed that the POA might predict the incidence of POD. However, the POA seems not to affect the duration and severity of the POD. The participants with POA were fragile after surgery due to the longer length of stay in hospital.

In order to clarify the relationship between POA and POD, the other factors, such as the emergency surgery and the history of cognitive impairment, that might interfere with the results had been strictly controlled in this case-study. Additionally, the alcohol and smoke use, education level, pain and the surgery time, which might affect the incidence of the POD, were recorded and showed no statistical differences.

The factor of POA predicted the incidence of POD in the study. In consistence with Saho’s study [22], they found that POA strongly predicted POD in cancer patients, which was confirmed our conclusion. The main difference between two studies was the time of anxiety. For the cancer patients usually had been threaten by death, they were more likely to be anxiety or even depressed after diagnosis. This situation lasted for a long time. However, the patients undergoing THA in our study had a few threats from death. They usually felt anxiety or stress in the day before surgery, even though they had endured pain when moved. On the other hand, Van Grootven reported the POA was not associated with the POD in a retrospective study of hip fracture patients [19]. Additionally, Detroyer also found no relationship between the two conditions in cardiac surgery patients [15]. However, the limitations were found in the studies, such as a retrospective study design [19] and the fact that the delirium evaluators were not psychiatric experts [15]. In this prospective study, every delirium case was confirmed by the skillful geriatric psychiatrist. Moreover, it was also proved in the multivariable logistic regression model, in which anxiety, age, alcohol abuse, education level and stroke history were detected as predictors that were also reported in some researches [26, 41, 42]. As anxiety was a quite common symptom before surgery. Our findings suggested that the POA may be a new indicator for the POD prophylaxis [22].

To explore the exact mechanism, we hypothesized that the inflammatory cytokines may be involved. It is reported that peripheral inflammatory cytokines migrate to the central nervous system and interact with microglia, causing neuroinflammation and the subsequent development of delirium [43–45]. Similarly, the pathway was also involved in the process of anxiety [46, 47], therefore, resulting from the increased release of inflammatory cytokines in central nervous system, the POA could be a good predictor for the POD.

There were several limitations in this study. First of all, the related mechanism was not explored during the trial. This study was aimed to confirm the relationship between the POA and the POD. The underlying mechanism will be investigated in our next step. Secondly, this is the single center trial with small sample size. A multi-center randomized controlled study was required to confirm the issue. Thirdly, the randomization was hard to performed in the study. As all participants should be categorized according to the HADS results. However, the control and the double blind was performed to reduce the bias in the study.

## Conclusions

In conclusion, the POA predicted the incidence of POD in patients undergoing total hip arthroplasty. The related intervention may be a good point for delirium prophylaxis.

## Data Availability

The datasets used and/or analyzed during the current study are available from the corresponding author for reasonable request. And the datasets will be available at www.chictr.org.cn in 6 months after publication.

## Abbreviations

HADS-A: The Hospital Anxiety and Depression Scale-Anxiety
CAM: The Confusion Assessment Method
MDAS: The Memorial Delirium Assessment Scale
OR: Odds Ratio
CI: Confidence Interval
POD: The Postoperative Delirium
POA: The Preoperative Anxiety
THA: The total Hip Arthroplasty
USTC: The University of Science and Technology of China
ChiCTR: The Chinese Clinical Trial Registry
AHPH: The Anhui Provincial Hospital
MMSE: The Mini-mental State Examination
LOS: Length Of Stay
BMI: Body Mass Index
IHD: Ischemic Heart Disease
ASA: American Society of Anesthesiologists
VAS: Visual Analogue Scale
SD: Standard Deviation
